# FENS‐Kavli winter symposium: Addressing the cellular phase of dementia—visions of the UK Dementia Research Institute

**DOI:** 10.1111/ejn.14059

**Published:** 2018-07-21

**Authors:** Raphael Hesse, Christopher M. Henstridge

**Affiliations:** ^1^ Centre for Discovery Brain Sciences UK Dementia Research Institute The University of Edinburgh Edinburgh UK

**Keywords:** Alzheimer's, dementia, FENS, Kavli

## Abstract

This is a short report summarising the plenary talk by Professor Bart de Strooper, at the 2017 FENS‐Kavli winter symposium. We have tried to capture some of the key points in his lecture on dementia research and we discuss his vision for the new UK Dementia Research Institute (UK DRI). In his talk, Prof. de Strooper encourages us to focus on the multicellular influence on brain dysfunction in dementia and we summarise how the UK DRI is a timely and ambitious, collaborative endevour, aiming to conquer dementia.

Alzheimer's disease (AD) is one of mankind's greatest afflictions. It is the most common degenerative illness of the human nervous system and the leading form of dementia worldwide. Almost unknown to the community 40 years ago, AD affects approximately 1%–2% of the world's population, with ageing the highest risk factor. The neuropathological features of AD are mainly intracellular neurofibrillary tangles (NFTs) consisting of hyperphosphorylated tau protein and extracellular amyloid beta (Aβ) plaques (Mandelkow & Mandelkow, 1998; Serrano‐Pozo, Frosch, Masliah, & Hyman, 2011). However, the best correlate with clinical dementia is the characteristic neuronal and synaptic loss (DeKosky, Scheff, & Styren, 1996; Masliah & Terry, 1993; Scheff, Price, Schmitt, & Mufson, 2006; Terry et al., 1991).

Alzheimer's disease has numerous risk factors, approximately 70% of which are genetic and 30% have an environmental background (Dorszewska, Prendecki, Oczkowska, Dezor, & Kozubski, 2016). Rare, dominant mutations that cause familial AD are located either within the amyloid precursor protein (APP) or within one of the APP cleaving enzymes, such as presenilin 1 and 2 (PSEN1 and PSEN2). Based on these observations, Hardy & Allsop postulated that the primary events in AD pathogenesis are increased Aβ load as a consequence of altered APP metabolism and that tau phosphorylation and NFTs occur secondarily (Hardy & Allsop, 1991). This is known as the amyloid cascade hypothesis, which was later modified and “corrected” to describe a shift towards APP cleavage into larger Aβ42 fragments, rather than Aβ40, leading to increased plaque deposition.

However, we still have large gaps in our knowledge of disease mechanisms and we still have no disease‐modifying treatments. To increase our understanding of the disease, generate novel therapeutic strategies and ultimately help patients suffering from dementia, a new research unit was founded in 2016: the UK Dementia Research Institute (DRI). The Medical Research Council (MRC), Alzheimer's Society and Alzheimer's Research UK invested £250 million to help the UK DRI break new ground by bringing together world‐leading expertise in biomedical, care and translational dementia research.

Prof. Bart de Strooper is the head of the UK DRI and recently gave a plenary talk at the FENS‐Kavli Winter Meeting (5 December–8 December 2017) at the Institute of Science and Technology in Klosterneuburg, Austria. He opened the talk by highlighting Aβ as the culprit in AD pathogenesis, supported by the fact that all mutations in familial AD (causal genes) and many of the risk genes affect Aβ aggregation. More than 180 mutations have been found in the Aβ generating γ‐secretases, PSEN1 & PSEN2, which historically made them promising and intensely studied targets for potentially reducing Aβ accumulation in brains of AD patients. However, PSEN‐targeted drugs in phase III clinical trials failed to improve cognitive function and failed to significantly reduce Aβ burden (Doody et al., 2013). Relatively late in these studies, it became clear that side effects further limited the clinical use of PSEN inhibitors (De Strooper, 2014).

The question we should ask is, why did the trials fail? Prof. de Strooper highlighted an important point during his talk which may help us understand this, namely that PSEN1 possesses conformational flexibility which is largely influenced by single nucleotide polymorphisms (SNPs) in the PSEN1 gene (Bai, Rajendra, Yang, Shi, & Scheres, 2015). These SNPs cause a conformational change in the structure and activity of PSEN1; therefore, depending on the genetics of each AD patient, drugs targeting this enzyme will likely have differing effects. Moreover, PSEN1 does not possess a single nonsense mutation, meaning that all known PSEN1 mutations or SNPs likely alter PSEN1 function and in this way contribute to AD pathology and the patient's response to PSEN1‐targeted therapeutics. For example, some of the PSEN1 mutations destabilize the enzyme leading to the release of longer forms of Aβ (≥42) (Szaruga et al., 2015). Thus, Prof. de Strooper drew two conclusions, first, that qualitative changes in Aβ generation cause AD, and second, that these findings provide the theoretical basis for the development of γ‐secretase/substrate stabilizing compounds for the treatment of AD.

When summarizing what the clinical mutations really teach us, Prof. de Strooper concluded that we should aim to (pharmacologically) stabilize the enzyme, we should target only one of the PSEN enzymes and not all four at once, we should be mindful of enzyme kinetics and last but not least, we should not give up the best‐validated drug target for AD.

Despite these significant findings, Prof. de Strooper was cautious in describing malfunction of PSEN1 as causative for AD, suggesting that the numerous studies on AD genetics provide powerful insights into pathomechanisms beyond PSEN1 failure. Special emphasis was put on changes in (phospho)lipid metabolism and microglial dysfunction. Bridging these findings, recent AD research has shown more and more clearly that AD is a chronic multicellular disorder and not solely a breakdown of neurons and synapses. The amyloid hypothesis has been refined over the last 25 years, but still falls short of fully explaining AD pathogenesis (Karran & De Strooper, 2016). Whilst some genetic studies highlight the role of Aβ42 in disease pathogenesis, the neuron‐centric view and the linearity of the cascade remain under debate (De Strooper & Karran, 2016). Furthermore, many of the genetic risk factors involve genes highly expressed in glial cells rather than neurons (Karch & Goate, 2015). Taken together, there is an urgent need to increase our understanding of the multicellular aspects of AD and discover the roles glial cells undoubtedly play in AD pathogenesis.

The question Prof. de Strooper raised was how to design good quality experiments to examine the multicellular shape of AD. He suggested using an APP mouse model, presenting both Aβ plaque deposition and glial activation. He described the APP knock‐in model, which harbours the Swedish, the Iberian/Beyreuther and the Arctic mutations in the humanized endogenous mouse APP gene. These mice have no drawbacks of overexpression and therefore no aberrant accumulation of other APP cleavage products. They also express APP under the endogenous promotor meaning it should be expressed in the right cells at the right time. Furthermore, this model shows progressive astro‐ and microgliosis in an age‐dependent manner, thus merging important hallmarks of AD (Masuda et al., 2016; Saito et al., 2014). Using this mouse model, Prof. de Strooper's group prepared cortex and hippocampus cell suspensions from C56/Bl6 and APP^KI^ mice, isolated 10,000 single microglia and analysed up to 3,000 genes at four different time points (3, 6, 12 and 21 months). By doing this, his team can identify genetic and protein changes in individual cell types, rather than in a whole brain homogenate where all genes and proteins from all cells are combined into one sample. As Prof. de Strooper put it: “We want fruit, not smoothies.” The analysis of this exciting study is still ongoing but promises to reveal valuable insights into disease progression with a focus on the multicellular nature of AD.

Another important point to address is how human cells react in AD. Whilst it is currently beyond our technology to accurately study changes in the living human brain at the cellular level, Prof. de Strooper has come up with a fascinating alternative. A study by Espuny‐Camacho and Arranz et al. described transplanting iPSC‐derived human neurons into an APP/PS1 mouse to develop a chimeric AD animal that contains implanted human neurons. Analysing the human–mouse chimera, they noticed significant degeneration and loss of human neurons with significant tau pathology (although no tangle formation), which was absent when these neurons were injected into a wild‐type mouse. A genomewide expression analysis of the reisolated human neurons showed upregulation of genes involved in myelination and downregulation of genes related to memory and cognition, synaptic transmission and neuronal morphology (Espuny‐Camacho et al., 2017).

This study suggests a clear non‐cell‐autonomous influence on human neuronal survival in the AD mouse brain. It would be interesting to know how other human cells would react to this implantation. For example, does the toxic environment of the AD mouse brain lead to death or phenotypic change in implanted human microglia? These questions were raised by Prof. de Strooper and work is underway in his laboratory to address them.

Given the emerging focus on AD as a non‐cell‐autonomous disorder, with influence from virtually all cell types in the brain, the UK DRI is incredibly well placed to significantly advance this area of research and reveal novel insights into disease pathogenesis. The UK DRI will unite 400 researchers from six universities in three nations (England, Scotland and Wales) with a shared vision: addressing the cellular phase of dementia. The central UK DRI hub is based at University College London, with five DRI centres located in University of Cambridge, Cardiff University, University of Edinburgh, Imperial College London and King's College London. Their mission is to revolutionize dementia research by finding new ways to diagnose the disease, improve quality of life for patients and most ambitiously of all, to prevent dementia. Prof. de Strooper wants to build on strong creativity and collaboration within and across DRI centres, leading to international excellence and recognition. The UK DRI will also facilitate international collaborations. Of note, UK DRI researchers already have collaborative publications with other FENS‐Kavli Network of Excellence scholars examining synapse degeneration in dementia models (McInnes et al., 2018; Zhou et al., 2017).

The UK DRI centre at the University of Edinburgh focuses on this question of multicellular influence on AD progression, highlighting the interplay between several cell types in the brain leading to neuron loss. As part of the research team in Edinburgh, we will investigate the complex interaction between neurons, glial cells and blood vessels, thereby creating a holistic approach to address the cellular phase of AD in order to pave the way for future clinical trials.

## References

[ejn14059-bib-0001] Bai, X. C. , Rajendra, E. , Yang, G. , Shi, Y. , & Scheres, S. H. (2015). Sampling the conformational space of the catalytic subunit of human γ‐secretase. Elife, 4, pii: e11182. 10.7554/eLife.11182 PMC4718806 26623517

[ejn14059-bib-0002] De Strooper, B. (2014). Lessons from a failed γ‐secretase Alzheimer trial. Cell, 159, 721–726. 10.1016/j.cell.2014.10.016 25417150

[ejn14059-bib-0003] De Strooper, B. , & Karran, E. (2016). The cellular phase of Alzheimer's disease. Cell, 164, 603–615. 10.1016/j.cell.2015.12.056 26871627

[ejn14059-bib-0004] DeKosky, S. T. , Scheff, S. W. , & Styren, S. D. (1996). Structural correlates of cognition in dementia: Quantification and assessment of synapse change. Neurodegeneration, 5, 417–421. 10.1006/neur.1996.0056 9117556

[ejn14059-bib-0005] Doody, R. S. , Raman, R. , Farlow, M. , Iwatsubo, T. , Vellas, B. , Joffe, S. , … Semagacestat Study Group (2013). A phase 3 trial of semagacestat for treatment of Alzheimer's disease. New England Journal of Medicine, 369, 341–350. 10.1056/NEJMoa1210951 23883379

[ejn14059-bib-0006] Dorszewska, J. , Prendecki, M. , Oczkowska, A. , Dezor, M. , & Kozubski, W. (2016). Molecular basis of familial and sporadic Alzheimer's disease. Current Alzheimer Research, 13, 952–963. 10.2174/1567205013666160314150501 26971934

[ejn14059-bib-0007] Espuny‐Camacho, I. , Arranz, A. M. , Fiers, M. , Snellinx, A. , Ando, K. , Munck, S. , … De Strooper, B. (2017). Hallmarks of Alzheimer's disease in stem‐cell‐derived human neurons transplanted into mouse brain. Neuron, 93, 1066–1081.e1068. 10.1016/j.neuron.2017.02.001 28238547

[ejn14059-bib-0008] Hardy, J. , & Allsop, D. (1991). Amyloid deposition as the central event in the aetiology of Alzheimer's disease. Trends in Pharmacological Sciences, 12, 383–388. 10.1016/0165-6147(91)90609-V 1763432

[ejn14059-bib-0009] Karch, C. M. , & Goate, A. M. (2015). Alzheimer's disease risk genes and mechanisms of disease pathogenesis. Biological Psychiatry, 77, 43–51. 10.1016/j.biopsych.2014.05.006 24951455 PMC4234692

[ejn14059-bib-0010] Karran, E. , & De Strooper, B. (2016). The amyloid cascade hypothesis: Are we poised for success or failure? Journal of Neurochemistry, 139(Suppl. 2), 237–252. 10.1111/jnc.13632 27255958

[ejn14059-bib-0011] Mandelkow, E. M. , & Mandelkow, E. (1998). Tau in Alzheimer's disease. Trends in Cell Biology, 8, 425–427. 10.1016/S0962-8924(98)01368-3 9854307

[ejn14059-bib-0012] Masliah, E. , & Terry, R. (1993). The role of synaptic proteins in the pathogenesis of disorders of the central nervous system. Brain Pathology, 3, 77–85. 10.1111/j.1750-3639.1993.tb00728.x 8269086

[ejn14059-bib-0013] Masuda, A. , Kobayashi, Y. , Kogo, N. , Saito, T. , Saido, T. C. , & Itohara, S. (2016). Cognitive deficits in single App knock‐in mouse models. Neurobiology of Learning and Memory, 135, 73–82. 10.1016/j.nlm.2016.07.001 27377630

[ejn14059-bib-0014] McInnes, J. , Wierda, K. , Snellinx, A. , Bounti, L. , Wang, Y. C. , Stancu, I. C. , … Verstreken, P. (2018). Synaptogyrin‐3 mediates presynaptic dysfunction induced by tau. Neuron, 97(4), 823–835. 10.1016/j.neuron.2018.01.022 29398363

[ejn14059-bib-0015] Saito, T. , Matsuba, Y. , Mihira, N. , Takano, J. , Nilsson, P. , Itohara, S. , … Saido, T. C. (2014). Single App knock‐in mouse models of Alzheimer's disease. Nature Neuroscience, 17, 661–663. 10.1038/nn.3697 24728269

[ejn14059-bib-0016] Scheff, S. W. , Price, D. A. , Schmitt, F. A. , & Mufson, E. J. (2006). Hippocampal synaptic loss in early Alzheimer's disease and mild cognitive impairment. Neurobiology of Aging, 27, 1372–1384. 10.1016/j.neurobiolaging.2005.09.012 16289476

[ejn14059-bib-0017] Serrano‐Pozo, A. , Frosch, M. P. , Masliah, E. , & Hyman, B. T. (2011). Neuropathological alterations in Alzheimer disease. Cold Spring Harbor Perspectives in Medicine, 1, a006189. 22229116 10.1101/cshperspect.a006189 PMC3234452

[ejn14059-bib-0018] Szaruga, M. , Veugelen, S. , Benurwar, M. , Lismont, S. , Sepulveda‐Falla, D. , Lleo, A. , … Chávez‐Gutiérrez, L. (2015). Qualitative changes in human γ‐secretase underlie familial Alzheimer's disease. Journal of Experimental Medicine, 212, 2003–2013. 10.1084/jem.20150892 26481686 PMC4647268

[ejn14059-bib-0019] Terry, R. D. , Masliah, E. , Salmon, D. P. , Butters, N. , DeTeresa, R. , Hill, R. , … Katzman, R. (1991). Physical basis of cognitive alterations in Alzheimer's disease: Synapse loss is the major correlate of cognitive impairment. Annals of Neurology, 30, 572–580. 10.1002/(ISSN)1531-8249 1789684

[ejn14059-bib-0020] Zhou, L. , McInnes, J. , Wierda, K. , Holt, M. , Herrmann, A. G. , Jackson, R. J. , … Verstreken, P. (2017). Tau association with synaptic vesicles causes presynaptic dysfunction. Nature Communications, 8, 15295 10.1038/ncomms15295 PMC5437271 28492240

